# A Novel Combination of Vitamin C, Curcumin and Glycyrrhizic Acid Potentially Regulates Immune and Inflammatory Response Associated with Coronavirus Infections: A Perspective from System Biology Analysis

**DOI:** 10.3390/nu12041193

**Published:** 2020-04-24

**Authors:** Liang Chen, Chun Hu, Molly Hood, Xue Zhang, Lu Zhang, Juntao Kan, Jun Du

**Affiliations:** 1 Nutrilite Health Institute, 720 Cailun Road, Shanghai 201203, China; clark.chen@amway.com (L.C.); iris.zhang@amway.com (X.Z.); lynn.zhang@amway.com (L.Z.); Junot.kan@amway.com (J.K.); 2 Nutrilite Health Institute, 5600 Beach Boulevard, Buena Park, CA 90621, USA; charles.hu@amway.com; 3 Nutrilite Health Institute, 7575 East Fulton Avenue, Ada, MI 49355, USA; molly.hood@amway.com

**Keywords:** coronavirus, vitamin C, curcumin, glycyrrhizic acid, system biology, inflammatory response, immune response

## Abstract

Novel coronaviruses (CoV) have emerged periodically around the world in recent years. The recurrent spreading of CoVs imposes an ongoing threat to global health and the economy. Since no specific therapy for these CoVs is available, any beneficial approach (including nutritional and dietary approach) is worth investigation. Based on recent advances in nutrients and phytonutrients research, a novel combination of vitamin C, curcumin and glycyrrhizic acid (VCG Plus) was developed that has potential against CoV infection. System biology tools were applied to explore the potential of VCG Plus in modulating targets and pathways relevant to immune and inflammation responses. Gene target acquisition, gene ontology and Kyoto encyclopedia of genes and genomes (KEGG) pathway enrichment were conducted consecutively along with network analysis. The results show that VCG Plus can act on 88 hub targets which are closely connected and associated with immune and inflammatory responses. Specifically, VCG Plus has the potential to regulate innate immune response by acting on NOD-like and Toll-like signaling pathways to promote interferons production, activate and balance T-cells, and regulate the inflammatory response by inhibiting PI3K/AKT, NF-κB and MAPK signaling pathways. All these biological processes and pathways have been well documented in CoV infections studies. Therefore, our findings suggest that VCG Plus may be helpful in regulating immune response to combat CoV infections and inhibit excessive inflammatory responses to prevent the onset of cytokine storm. However, further in vitro and in vivo experiments are warranted to validate the current findings with system biology tools. Our current approach provides a new strategy in predicting formulation rationale when developing new dietary supplements.

## 1. Introduction

Coronaviruses (CoVs) belong to the *Coronaviridae* virus family and are enveloped, positive-sense RNA viruses [1]. CoVs infect various host species, including humans and other vertebrates. In recent years, novel CoVs emerged periodically in different regions around the globe, such as severe acute respiratory syndrome CoV (SARS-CoV) in 2002, Middle East respiratory syndrome CoV (MERS-CoV) in 2012 and SARS-CoV-2 in late 2019 [2]. These viruses predominantly cause respiratory and intestinal tract infections and induce various clinical manifestations [3]. Although the pathologies of these virus are not yet completely understood, viral proteins and host factors play key roles in the infection process [4]. A well-coordinated immune response is essential against virus infection. In contrast, an out of control immune response is associated with immunopathogenesis and excessive inflammatory response, which may result in poor outcomes such as severe pulmonary damage and multi-organ failure [5,6]. Due to the challenges of developing antiviral drugs and vaccines, the outbreaks of CoV infections often cause major public health issues [7]. CoV-infected people must rely on their own immune defense to control the progress of infection. These diseases are classified as self-limiting diseases, meaning that an individual’s immune function will determine whether early symptoms will advance into severe acute respiratory tract symptoms (i.e., pneumonia) or recovery from infection. 

Phytonutrients are a variety of bioactive non-nutrient plant compounds that exhibit the capacity to alter biochemical reactions and consequently influence human health after ingestion [8,9]. Commonly known phytonutrients in dietary supplements include flavonoids, anthocyanin, carotenoids, polyphenols, triterpenoids and phytosterols, many of which have been reported to play important roles in human health with potential as therapeutic agents [10,11]. It is well-known that adequate intake of nutrients and phytonutrients may help regulate immune function, including enhancing defense and resistance to infection, while maintaining tolerance [12]. Several plant food sources, such as acerola berry (*Malpighia glabra* L., *M. emarginata* D.C.), roxburgh rose fruit (*Rosa roxburghii* Tratt.), camu camu (*Myrciaria dubia* (Kunth) McVaugh), amla (*Phyllanthus emblica* L.) and sea buckthorn berry (*Hippophae rhamnoides* L.) are known as rich sources of vitamin C (VC). VC regulates immunity by enhancing differentiation and proliferation of B- and T-cells, and it is beneficial in preventing and treating respiratory and systemic infections [13,14,15]. VC potentially protects against infection caused by CoVs due to its benefits on immune function [16]. High doses of VC were recommended for prevention of SARS-CoV-2 infections by the Chinese Center for Disease Control and Prevention and Chinese Nutrition Society. Currently, VC is under investigation in a clinical trial for its benefit in patients with severe SARS-CoV-2 infection (https://clinicaltrials.gov/). 

Glycyrrhizic acid (GA) is a major phytonutrient found in licorice root (*Glycyrrhiza uralensis* Fisch. ex DC., *G. inflata* Bat., *G. glabra* L.), which is considered an ingredient for both food and medicinal use in China [17]. GA exhibits anti-viral [18], anti-inflammatory [19] and hepatoprotective activities [20]. Traditional Chinese medicine (TCM) treatments for SARS-CoV-2 infection pneumonia were recommended by National Health Commission of China, and licorice root was one of the commonly used TCM herbs [21]. GA has been reported recently for its binding capability with angiotensin-converting enzyme 2 (ACE2) to prevent SARS-CoV-2 infection [22]. Intriguingly, the effect of diammonium glycyrrhizinate combined with vitamin C tablets on common pneumonia infected with SARS-CoV-2 is being tested in clinical trials (http://www.chictr.org.cn/). 

Curcumin (CC) and its analogues are the main phytonutrients of turmeric (*Curcuma longa* L.) and other *Curcuma* spp., which are widely used around the world as culinary spices, traditional medicine as well as a popular dietary supplement ingredient due to its wide range of health benefits including anti-inflammation [23], anti-cancer [24], cardiovascular regulation [25], respiratory [26] and immune system benefits [27]. In addition, the suppression of multiple cytokines by curcumin suggested that it may be a useful approach in treating Ebola patients against cytokine storm [28]. CC also inhibited aminopeptidase N (APN) which was identified as a cellular receptor for alpha CoV [29]. 

Since VC, CC and GA are popular in nutrition, and more importantly, they have been used to regulate immune responses and recommended to intervene in CoV infections, a combination of VC, CC and GA (VCG Plus) was proposed for its potential to prevent CoVs infection. In the present study, our objective is to apply system biology techniques to investigate biological processes and pathways that are regulated by VCG Plus, and to illustrate how these biological processes and pathways could be associated with protection against CoV infections. 

## 2. Method

### 2.1. Gene Target Acquisition and Screening

Comprehensive determination of potential compound–target interaction profiles is a critical step for the system biology analysis [30]. Currently, multiple databases/platforms, such as DrugBank Database, Comparative Toxicogenomics Database (CTD), Traditional Chinese Medicine Systems Pharmacology Database and Analysis Platform (TCMSP) and Integrative Pharmacology-based Research Platform of Traditional Chinese Medicine (TCMIP), were commonly applied to acquire potential targets of small molecular compounds [31,32,33]. DrugBank contains detailed drug, drug-target, drug action and drug interaction information about FDA-approved drugs as well as experimental drugs [34]. CTD provides core information on chemical-gene interactions that are manually curated from scientific literature [35,36]. While TCMIP predicts the potential targets for herbal chemical compounds using MedChem Studio (version 3.0), an efficient drug similarity search tool to identify herbal chemical compounds with high structural similarity (Tanimoto score > 0.8) to known drugs [37]. Basically, the target information in these three databases is complementary, a combination of which could provide relatively comprehensive compound-target interactions. In this work, the target acquisition of VC, CC and GA was conducted separately, using direct text mining of DrugBank, CTD and TCMIP with their chemical names as keywords. The targets of VC and CC from CTD with interaction counts less than 5 were excluded. All acquired targets of VC, CC and GA were limited to *Homo sapiens* and mapped to UniProt [38] for correction to remove redundant and erroneous ones.

### 2.2. Hub Target Identification and Protein–Protein Interaction (PPI) Analysis

Hub targets were identified by taking following steps: 

(1) Combine the targets of VC, CC and GA and remove the duplicates; 

(2) Map them into the CTD website, choose “virus diseases” and “immune system diseases” gene database for comparison, select the overlapping targets for the next analysis; 

(3) Map selected targets into STRING (Version 11.0) to perform PPI analysis [39], set the cut-off degree of PPI as high confidence (0.700), and download the information of PPI as TSV file format; 

(4) Import the file to Cytoscape software (Version 3.6.1) [40] to analyze the topological parameters of the interactions, select the hub targets whose node degree is greater than the median value. After these steps, STRING and Cytoscape are used subsequently to construct and analyze the PPI network of hub targets. In constructed networks, the targets are represented by nodes while the interactions among them are represented by edges.

### 2.3. Distribution Analysis of Targets in Tissues/System and Gene Ontology (GO) Enrichment and Analysis 

Gene ORGANizer [41] was employed to perform the target-system location analysis. DAVID Bioinformatics Resources 6.8 [42] was applied to perform GO analysis for the hub targets. The biological process, cell component and molecular function were three basic outputs of GO. The cut-off value of the *p*-value was set to 0.05, and the *p*-value was adjusted using the Benjamini–Hochberg method. In addition, the analysis of specific GO annotation involved in immune system processes was carried out with ClueGo (Version 2.5.6) [43], a Cytoscape plug-in integrating EBI-Uniport GO annotation database (updated in Mar 2019). Generally, the targets from VC, CC and GA were imported to ClueGo separately and represented by different colors. The visual style of ClueGo analysis was set as “cluster”. The GO term/pathway was added to a specific cluster term if at least 80% of genes in this term is contributed by an individual (phyto-) nutrient. Only terms with a *p*-value less than 0.05 were presented after two-side hypergenometric test and bonferroni step down adjustment were conducted. 

### 2.4. Kyoto Encyclopedia of Genes and Genomes (KEGG) Pathway Analysis 

KEGG pathway enrichment and analysis were performed on ClueGo integrating with KEGG database (updated in 17 February 2020). The procedures were similar to the immune system process GO term analysis, briefly described below: 

(1) import the targets of VC, CC and GA to ClueGo separately, represent by different colors;

(2) set visual style as “cluster”, and set statistical method as two-side hypergenometric test and bonferroni step down adjustment, only pathways with *p*-value less than 0.05 are shown; 

(3) start analysis, download the protein-pathway interactions information in Excel format for analysis. According to KEGG database, pathways are clustered into the following categories: (A) metabolism, (B) genetic information processing, (C) environmental information processing, (D) cellular processes, (E) organismal systems, and (F) human diseases. Finally, the top 15 protein–pathway interactions related to immune and inflammatory responses were extracted and shown. 

## 3. Results

### 3.1. Hub Target Identification and Analysis 

**Figure 1:**
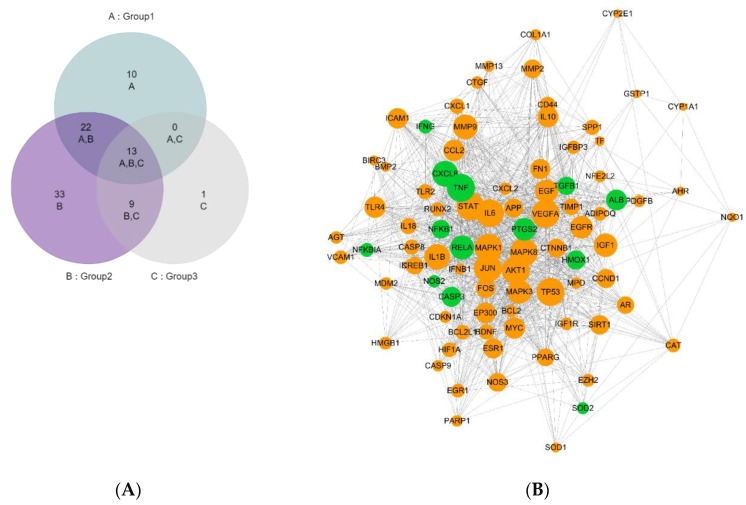
Hub target analysis of VCG Plus. A Venn diagram of hub target distribution in VC, CC and GA, respectively (**A**). PPI network of 88 hub targets of VCG Plus (**B**). OmicsBean (http://www.omicsbean.cn/) was employed to draw Figure 1A. Cytoscape software (Version 3.6.1) was employed to draw Figure 1B. In Figure 1B, all the targets are represented by nodes, whereas the interaction between the targets are represented by edges. The node size is proportional to the node degree. The intersect targets of VC, CC and GA are represented by green. VCG Plus, the combination of vitamin C, curcumin and glycyrrhizic acid. VC, vitamin C (group1); CC, curcumin (group 2); GA, glycyrrhizic acid (group 3). PPI, protein-protein interaction.

Three public databases were used to mine the potential targets for the three (phyto-) nutrients in VCG Plus. The number of qualified targets identified for VC, CC and GA were 109, 146, and 65, respectively (Supplementary Table S1), and a total of 248 unique targets were identified for the combination of VCG Plus (phyto-) nutrients. Comparing the results with “virus diseases” and “immune system disease” gene data in CTD, it was found that 179 targets existed in both the “virus diseases” and “immune system disease” gene database. These 179 targets were then selected to perform PPI analysis and network topological analysis. As a result, 88 tightly connected targets (hub targets, node degree ≥ 12) were identified for further analysis. Detailed information of the 88 hub targets is shown in Table 1. A Venn diagram (Figure 1A) shows that 13 targets overlap for the combination of VCG Plus (phyto-) nutrients, which include ALB, CASP3, CXCL8, HMOX1, NFKB1, NFKBIA, PTGS2, RELA, TGFB1 NOS2, SOD2, IFNG and TNF. In addition, there are nine overlapping targets for CC and GA, and 22 overlapping targets for VC and CC. Furthermore, the PPI of hub targets was constructed by STRING and they are shown in Figure 1B. The PPI network was assembled by 88 nodes (targets) and 1153 edges (interactions), with clustering coefficients of 0.59 and an average number of neighbors of 26.21. The targets are closely connected, suggesting that they may position in similar biological pathways with similar health benefits. 

### 3.2. Enrichment and Analysis of Target Distribution in Tissues and Systems 

**Figure 2:**
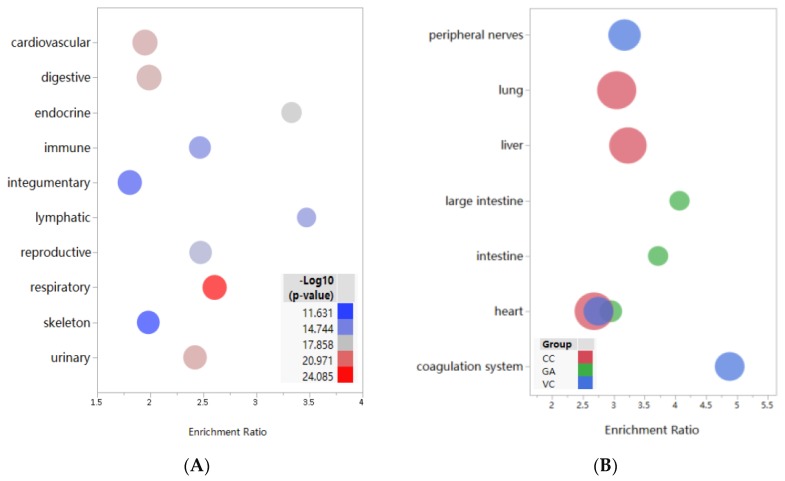
Distribution analysis of targets in tissues and systems. The bubble plots were made using JMP software 14.2.0 (SAS institute Inc. USA). Distribution of targets of VCG Plus in system (**A**), distribution of targets of VC, CC and GA in tissues (**B**). In Figure 2A, the bubble size is proportional to the targets number, and the shade of bubble is inversely proportional to the *p*-value. In Figure 2B, the bubble size is proportional to the targets number. The targets distribution of VC is represented by blue bubble, CC is represented by red bubble, and GA are represented by green bubble. VC, vitamin C; CC, curcumin; GA, glycyrrhizic acid.

We analyzed the system distribution of 88 targets to better explore the potential function on a system level. The top 10 systems are shown in Figure 2A. The respiratory system was found as the most significant location which contained 78 targets, followed by the urinary (74 targets), cardiovascular (84 targets), digestive (83 targets) and immune systems (64 targets). In addition, the tissue distribution of the targets for each (phyto-) nutrient was analyzed. The top three significant tissues of each individual compound were shown in Figure 2B. It is interesting that targets of these (phyto-) nutrients are all enriched in the heart. However, targets of CC are also enriched in the lung and liver, while targets of GA are enriched in the intestine and large intestine, and targets of VC are enriched in the peripheral nerves and coagulation system. 

CoV infections may lead to inflammation and alter immune responses, which are generally associated with the respiratory and immune systems [4,44]. Some digestive and cardiovascular events, such as diarrhea [45], heart palpitations [46] and abnormal coagulation parameters [47] were observed in clinical studies, suggesting that coronavirus infection may result in systemic damage. In this sense, the VCG Plus targets could cover most systems and tissues, indicating the potential to systematically intervene in the process of virus infection. The results also indicate that VCG Plus may have the potential to improve systematic immune and inflammatory responses caused by virus infections. 

### 3.3. Enrichment and Analysis of GO Term 

**Figure 3:**
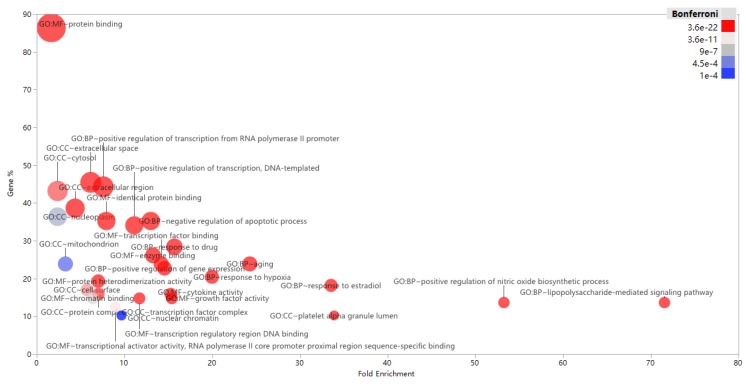
Top 10 gene ontology (GO) terms of biologic process, molecular function and cellular component, respectively. The bubble plot was made using JMP software 14.2.0 (SAS institute Inc. USA). The bubble size is proportional to the targets number, and the shade of bubble is inversely proportional to the *p*-value.

All enriched GO terms are available in Supplementary Table S2. The top 10 significant terms in biological process, molecular function and cellular component categories, respectively, are shown in Figure 3. VCG Plus is active in regulating transcription from RNA polymerase II promoter and transcription of DNA-templated via binding of transcription factor and chromatin. VCG Plus regulates the apoptotic process, nitric oxide biosynthetic process and lipopolysaccharide-mediated signaling pathway through cytokine activity, enzyme binding and/or protein binding. The biological process result for responding to hypoxia is worth mentioning, since a decline in oxygen saturation is commonly observed in SARS-CoV-2 infected patients [45]. The hypoxic response is a systemic process that regulates multiple cellular activities to maintain homeostasis under hypoxic condition [48]. In the present work, we note that both VC and CC could act on hypoxia inducible factor 1 alpha subunit (HIF-1A), suggesting their potential benefits on maintaining homeostasis under hypoxic conditions.

**Figure 4:**
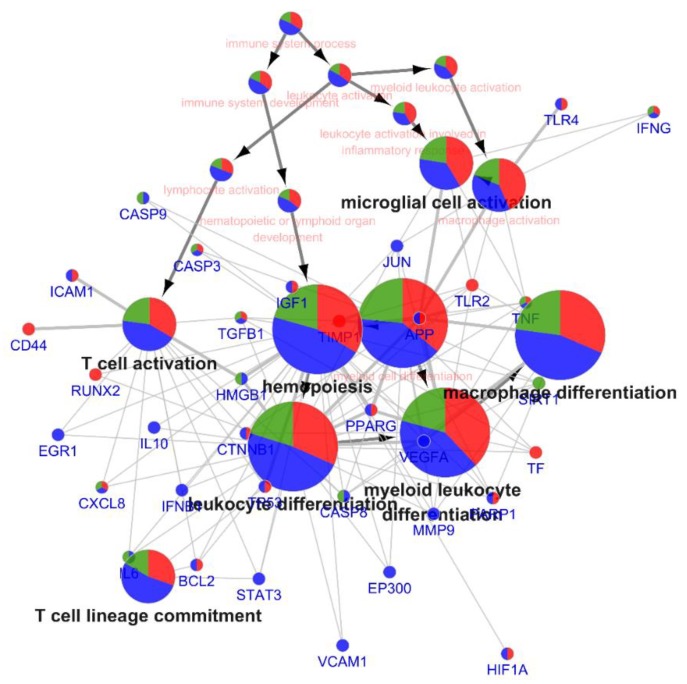
Target immune-related biologic process network. The network was constructed by ClueGo (Latest Version 2.5.6), integrating immune process EBI-Uniport GO annotation database. Only pathways with *p* < 0.05 are shown. The targets and biologic processes are represented by nodes while the interactions among them are represented by edges. Contribution of VC (vitamin c) in targets and pathways is represented by red, while CC (curcumin) is represented by blue, and GA (glycyrrhizic acid) is represented by green.

In addition, GO analysis of biological processes related to the immune system was performed using ClueGo. ClueGo was used to generate the targets-processes network of VC, CC and GA and shown as clusters, so that the role of each nutrient contributing to pathway regulation could be visualized (Figure 4). As a result, nine significant immune system processes were obtained, including differentiations of macrophage, leukocyte, myeloid cell and myeloid leukocyte, activation of macrophage and T-cell, T cell lineage commitment and hemopoiesis. These results suggest that VCG Plus may enhance immunity by modulating the regulation of immune cell differentiation and activation.

### 3.4. KEGG Pathway Enrichment and Analysis 

**Figure 5:**
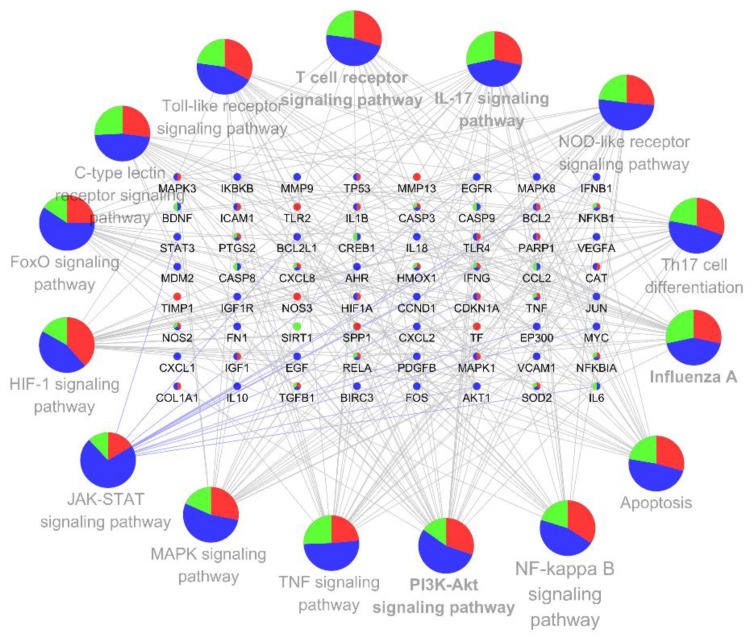
Target KEGG pathways network of VCG Plus. The network was constructed by ClueGo (Latest Version 2.5.6), integrating the latest KEGG pathway database. The targets and pathways are represented by nodes while the interactions among them are represented by edges. Contribution of VC (vitamin c) in targets and pathways is represented by red, while CC (curcumin) is represented by blue, and GA (glycyrrhizic acid) is represented by green.

All 88 identified targets were imported to ClueGo for KEGG pathway enrichment, resulting in 110 statistically significant pathways (Supplementary Table S3). According to the KEGG database, the obtained pathways are mainly concentrated on categories of signal transduction involved in environmental information processes, immune systems involved in organismal systems, infectious diseases involved in human diseases and other pathways. The top 15 pathways which are closely related to immunity, inflammation and RNA virus infections, along with effective target interactions were demonstrated in Figure 5. PI3K-AKT signaling pathway is associated with the most targets (30 targets), followed by TNF signaling pathway (25 targets), HIF-1 signaling pathway (23 targets), IL-17 signaling pathway (22 targets), NOD-like receptor signaling pathway (22 targets), Influenza A (21 targets), FoxO signaling pathway (20 targets), Toll-like receptor signaling pathway (19 targets), NF-κB signaling pathway (17 targets) and T helper (Th)17 cell differentiation (16 targets). Other pathways which belong to the immune system include T-cell receptor, Th17 cell differentiation and C-type lectin receptor signaling, and inflammation-related pathways including JAK-STAT signaling and apoptosis are also shown. 

## 4. Discussion

The interaction between CoV spike (S) protein and its receptor is the primary determinant for such virions attachment to human cells [49]. Multiple peptidases have been well described as CoV cellular receptors, including APN as the receptor for alpha CoV, angiotensin-converting enzyme 2 (ACE2) as the receptor for SARS-CoV and dipeptidyl-peptidase 4 (DPP4) as the receptor for MERS-CoV [1]. Inhibitors of S protein binding to receptor is a strategy for preventing and treating infection [7,50]. Although our data did not show that VCG Plus (phyto-) nutrients act on CoV cellular receptor, the potential capability of GA binding to ACE2 was reported recently [22]. Moreover, CC has been reported as the inhibitor of APN with potential to be a cancer chemoprevention agent [29]. The interactions between CC and APN, and GA and ACE2 were not included in our current analysis, mainly due to our strict rules for target screening. Through Venn analysis of targets from VCG Plus, silent mating type information regulation 2 homolog 1 (SIRT1) was found to only interact with GA. SIRT 1 belongs to the sirtuin family which contains seven proteins (SIRT1-7) that are class III NAD^+^-dependent histone deacetylases (HDACs) [51]. It is interesting that SIRT1 has been shown to play both pro-viral and anti-viral roles, depending on the type of virus. The SIRT1 inhibitor showed a suppressive effect on hepatitis B virus (HBV) replication [51,52], while the SIRT1 activator showed a suppressive effect on human T-cell leukemia virus type 1 (HTLV-1) [53] and MERS-CoV [54]. Han [55] found that SIRT1 inhibited viral RNA transcription and translation in enterovirus 71 (EV 71, a RNA virus)-infected human rhabdomyosarcoma (RD) cells. Based on these results, it is possible that SIRT 1 could be an antiviral for RNA virus infections like MERS-CoV and EV 71. Containing the key phytochemical GA, licorice is generally associated with detoxication in TCM [56], and exhibits antiviral effect [57,58,59]. Others have found that GA activates SIRT1 in diabetic *db/db* mice [60] and increases the expression of SIRT1 in renal tubular epithelial cell line [61]. Hence, it is speculated that GA may exert anti-CoV effects via regulating SIRT 1 protein. However, further experimental research is needed to clarify the antivirus mechanism of GA as well as the role of SIRT1 in various CoV infections. 

The innate immune system is the first line of defense against virus infection. A rapid and well-coordinated innate immune response to sense invading viruses, and subsequent signal transduction pathways targeted to inhibit infection [62]. During a viral infection, host pathogen-recognition receptors (PRRs) initially sensitized by viral pathogen-associated molecular patterns and cascades of signaling pathways are activated to produce type 1 interferons (IFNs). IFNs are the prominent cytokines in innate immune response, and are thought to enhance the release of antiviral proteins for the protection of uninfected cells [5,63]. CoV can be sensed by three types of PRR, including Toll-like receptors, retinoic acid-inducible gene I (RIG-I)-like receptors, and nucleotide-binding and oligomerization domain (NOD)-like receptors [4]. Sometimes, accessory proteins of SARS-CoV and MERS-CoV can interfere with PRRs, antagonize IFNs’ response and evade the immune response. The delayed IFNs’ response may result in uncontrolled inflammatory response [64,65]. In our present study, the results demonstrate the involvement of PRR signaling-related pathways including NOD-like receptors, Toll-like receptors (Figure 5) and RIG-I like receptors signaling (Supplementary Table S3) pathways in the biological functions of VCG Plus, as well as the IFNs (IFNG, IFNB1 in Table 1). Previous studies have revealed that CC significantly stimulated the production of IFN-β (IFNB1) in mice infected with influenza A virus (IAV), resulting in the increased survival rate and improvement of pulmonary histopathological changes [66]. Similarly, VC improved the production of IFN α/β (IFNA1/B1), activated anti-viral immune responses and remarkably increased the survival rate of VC-depleted mice infected with IAV [67,68]. In addition, multiple groups have demonstrated that GA improves IFN-γ (IFNG) production and ameliorates immune function [69,70,71]. These results indicate that VCG Plus may be beneficial in regulating innate immune response against invading viruses, through regulating NOD-like, Toll-like receptor signaling pathways, and promoting the production of IFNs.

T-cells, including CD4+ cells, and CD8+ cells play an antiviral role not only by combating against virions but also restricting the development of autoimmunity or overwhelming inflammation [4]. CD4+ cells promote the production of virus-specific antibodies via activating T-dependent B-cells, whereas CD8+ cells kill viral infected cells [72]. However, some CoVs are thought to induce T-cell apoptosis by the activation of apoptosis pathways [73], while depletion of CD4+ cells in later stages is associated with immune-mediated interstitial pneumonitis and delayed clearance of pathogen [74]. In SARS-CoV-2 infected patients, both the counts of CD4 + cells and CD8+ cells in severe pneumonia patients were lower than non-severe patients [75]. Similar results were observed in SARS-CoV infected patients [76,77]. In our current study, the significant interactions of VCG Plus related to immune cell differentiation and activation pathways were observed (Figure 4). The VCG Plus (phyto-) nutrients in this combination can co-regulate T-cell activation and other related biological processes by acting on different targets, suggesting the existence of a potential synergy. The literature has shown that VCG Plus (phyto-) nutrients positively regulate T-cells. For instance, VC positively influences lymphocyte development and function, and enhances T-cell proliferation and T-cell function [14,78]. CC could target regulatory T-cells and convert them into CD4+ Th1 cells to process anti-tumor effects [79,80], and improve the imbalance of Th1/Th2 subsets to process anti-inflammatory and anti-autoimmune effects [27,81]. GA showed anti-allergic effect by restoring the imbalance of Th1/Th2 subsets [82,83]. These results suggest that VCG Plus could promote the proliferation of Th1 cells and the production of virus-specific antibodies to compete CoV infections, and simultaneously regulate the Th1/Th2 subsets to prevent autoimmune and excessive inflammatory response in the later stage of infection. 

A cytokine storm, the massive overproduction of cytokines by the immune system, often appears in the terminal stage of some viral diseases (SARS, MERS, SARS-CoV-2). It is partially responsible for high fatality rates in patients infected with viruses [3]. In a cytokine storm, numerous pro-inflammatory cytokines such as IL-1, IL-6 and TNF-α, and inflammatory chemokines CCL3, CCL5, CCL2, and CXCL10 are released, leading to hypotension, hemorrhage, and eventually multiorgan failure [84]. MAPKs signaling [85], NF-κB signaling [86,87], TNF signaling [88] and PI3K/AKT signaling pathways [85,89], play important roles in mediating CoV infection-induced inflammatory responses. As a matter of fact, the anti-inflammatory effects of VC, CC and GA have been well documented. VC decreases IL-4, IL-6 and IL-8 level via inhibition of NF-κB signaling pathway in concanavalin A- induced liver injury mice [90]. Many studies have shown that CC presents anti-inflammatory function via NF-κB signaling [91,92], PI3K/AKT signaling [93], MAPK signaling [66] and TLRs signaling pathways [94]. In addition, GA alleviated inflammation via NF-kB and p38/ERK pathways in the reduction in multiple cytokines, including IL-6, TNF-α, IL-8, IL-1β and HMGB1 [95]. Consistently, the pathways mentioned above were successfully enriched and demonstrated in our result (Figure 5). Together with the evidence from the literature, our findings suggest that this combination may prevent the onset of cytokine storm. 

VC is an essential nutrient derived from plant sources, GA is derived from licorice, which is the most popular herb in TCM and other traditional medicine, and CC is derived from turmeric which is the most popular botanical source for Ayurveda medicine and culinary herbs. The combination of these three (phyto-) nutrients has not been reported previously, despite the single use of each ingredient has been widely studied. In this study, we first collected gene targets of VC, CC and GA, followed by target enrichment and analysis including distribution in tissues and systems, GO function and KEGG pathways. As target acquisition is the critical step for the whole analysis, an optimized strategy was used in our study. Briefly, we compared the targets from multiple databases, set high, reliable cut-off values and reviewed the text description of interactions, to ensure the high credibility of targets. In addition, we narrowed down the range by mapping to “immune system disease” and “virus diseases” related gene databases in CTD, to ensure a more focused analysis. After step by step system biology analysis, combined with up to date molecular mechanism investigations of CoV infections, our results suggest VCG Plus may regulate immune and inflammatory responses to prevent CoV infections by acting on multiple targets and pathways. Regulating NOD-like and Toll-like receptor signaling, promoting IFNs production, inhibition of PI3K/AKT, NF-κB and MAPK signaling, and activating and balancing T cells are the main functional mechanisms identified. In addition to the function of the individual (phyto-) nutrients in the VCG plus, they appear to be complementary and synergistic by modulating a variety of targets through similar or different signal pathways. 

There are limitations of the current investigation. For example, the pathogenic mechanism of CoV infection is not clearly understood yet, and the study of specific protections against CoV infections of VC, CC and GA was very limited. We only conducted the analysis on our best knowledge at the time. We started the analysis from known potential targets of VCG Plus, followed by enrichment analysis of biological processes and pathways which were generally associated with the immune system and viral infection. Based on the recent advances in the knowledge of CoV infection pathogenic mechanism and the findings from our analysis, VCG Plus regulates CoV infection pathways and were highlighted in our discussion. The results may not comprehensively illustrate how this combination would help immune system defense to CoV infections, but it demonstrates the potential of VCG Plus. In addition, the dose and route of administration of VCG or ADME were not taken into consideration in the current work. However, technologies to enhance bioavailability have been widely studied and indicated that advanced formulation processes could minimize these issues. Further in vitro mechanistic and preclinical studies are warranted in order to verify the directional prediction obtained from our current analysis. 

## 5. Conclusions

In summary, since no specific therapy for CoV infections is available, any potential way of protecting against CoV infections is worth studying and discussing. This paper investigated the potential protective effect of VCG Plus against CoV infections using systems biology. Our results suggest that VCG Plus is predicted to be helpful in regulating immune response against CoV infections and inhibiting excessive inflammatory response to prevent the onset of cytokine storm. However, further in vitro/in vivo experiments are warranted for validation. The analytical approach in this study provides a new thinking process to support the formulation strategy for the development of new dietary supplements with potential immune benefits. 

## Figures and Tables

**Table 1:** Hub targets identified for VCG Plus. VCG Plus, the combination of vitamin C, curcumin and glycyrrhizic acid. VC, vitamin C; CC, curcumin; GA, glycyrrhizic acid.

GENE_SYMBOL	Name	Distribution
EP300	E1A binding protein p300	CC only
VCAM1	vascular cell adhesion molecule 1	CC only
CCN2	cellular communication network factor 2	CC only
MYC	MYC proto-oncogene, bHLH transcription factor	CC only
VEGFA	vascular endothelial growth factor A	CC only
ADIPOQ	adiponectin, C1Q and collagen domain containing	CC only
IKBKB	inhibitor of nuclear factor kappa B kinase subunit beta	CC only
FN1	fibronectin 1	CC only
ESR1	estrogen receptor 1	CC only
MAPK8	mitogen-activated protein kinase 8	CC only
GSTP1	glutathione S-transferase pi 1	CC only
FOS	Fos proto-oncogene, AP-1 transcription factor subunit	CC only
AKT1	AKT serine/threonine kinase 1	CC only
IFNB1	interferon beta 1	CC only
MDM2	MDM2 proto-oncogene	CC only
CXCL1	C-X-C motif chemokine ligand 1	CC only
CXCL2	C-X-C motif chemokine ligand 2	CC only
PDGFB	platelet derived growth factor subunit B	CC only
AHR	aryl hydrocarbon receptor	CC only
CYP2E1	cytochrome P450 family 2 subfamily E member 1	CC only
EGFR	epidermal growth factor receptor	CC only
EGR1	early growth response 1	CC only
IGF1R	insulin like growth factor 1 receptor	CC only
BIRC3	baculoviral IAP repeat containing 3	CC only
IGFBP3	insulin like growth factor binding protein 3	CC only
STAT3	signal transducer and activator of transcription 3	CC only
EGF	epidermal growth factor	CC only
IL18	interleukin 18	CC only
CCND1	cyclin D1	CC only
MMP9	matrix metallopeptidase 9	CC only
BCL2L1	BCL2 like 1	CC only
JUN	Jun proto-oncogene, AP-1 transcription factor subunit	CC only
IL10	interleukin 10	CC only
HMGB1	high mobility group box 1	CC_GA_intersect
IL6	interleukin 6	CC_GA_intersect
CREB1	cAMP responsive element binding protein 1	CC_GA_intersect
IFNG	interferon gamma	CC_GA_intersect
BDNF	brain derived neurotrophic factor	CC_GA_intersect
MMP2	matrix metallopeptidase 2	CC_GA_intersect
CCL2	C-C motif chemokine ligand 2	CC_GA_intersect
CASP9	caspase 9	CC_GA_intersect
AR	androgen receptor	CC_GA_intersect
CASP8	caspase 8	CC_GA_intersect
SIRT1	silent mating type information regulation 2 homolog 1	GA only
BMP2	bone morphogenetic protein 2	VC only
TIMP1	TIMP metallopeptidase inhibitor 1	VC only
TLR2	toll like receptor 2	VC only
SPP1	secreted phosphoprotein 1	VC only
MMP13	matrix metallopeptidase 13	VC only
NOS3	nitric oxide synthase 3	VC only
TF	transferrin	VC only
RUNX2	RUNX family transcription factor 2	VC only
EZH2	enhancer of zeste 2 polycomb repressive complex 2 subunit	VC only
CD44	CD44 molecule	VC only
HMOX1	heme oxygenase 1	VC_CC_GA_intersect
RELA	RELA proto-oncogene, NF-κB subunit	VC_CC_GA_intersect
TGFB1	transforming growth factor beta 1	VC_CC_GA_intersect
PTGS2	prostaglandin-endoperoxide synthase 2	VC_CC_GA_intersect
NFKBIA	NF-κB inhibitor alpha	VC_CC_GA_intersect
NFKB1	nuclear factor kappa B subunit 1	VC_CC_GA_intersect
CXCL8	C-X-C motif chemokine ligand 8	VC_CC_GA_intersect
SOD2	superoxide dismutase 2, mitochondrial	VC_CC_GA_intersect
ALB	albumin	VC_CC_GA_intersect
TNF	tumor necrosis factor	VC_CC_GA_intersect
NOS2	nitric oxide synthase 2	VC_CC_GA_intersect
CASP3	caspase 3	VC_CC_GA_intersect
PARP1	poly (ADP-ribose) polymerase 1	VC_CC_intersect
CTNNB1	catenin beta 1	VC_CC_intersect
NQO1	NAD(P)H quinone dehydrogenase 1	VC_CC_intersect
NFE2L2	nuclear factor, erythroid 2 like 2	VC_CC_intersect
PPARG	peroxisome proliferator activated receptor gamma	VC_CC_intersect
IL1B	interleukin 1 beta	VC_CC_intersect
MAPK3	mitogen-activated protein kinase 3	VC_CC_intersect
MAPK1	mitogen-activated protein kinase 1	VC_CC_intersect
MPO	myeloperoxidase	VC_CC_intersect
TLR4	toll like receptor 4	VC_CC_intersect
COL1A1	collagen type I alpha 1 chain	VC_CC_intersect
AGT	angiotensinogen	VC_CC_intersect
APP	amyloid beta precursor protein	VC_CC_intersect
HIF1A	hypoxia inducible factor 1 alpha subunit	VC_CC_intersect
CDKN1A	cyclin dependent kinase inhibitor 1A	VC_CC_intersect
IGF1	insulin like growth factor 1	VC_CC_intersect
SOD1	superoxide dismutase 1	VC_CC_intersect
CYP1A1	cytochrome P450 family 1 subfamily A member 1	VC_CC_intersect
BCL2	BCL2, apoptosis regulator	VC_CC_intersect
TP53	tumor protein p53	VC_CC_intersect
CAT	catalase	VC_CC_intersect
ICAM1	intercellular adhesion molecule 1	VC_CC_intersect

## Supplementary Materials

The following are available online at https://www.mdpi.com/2072-6643/12/4/1193/s1, Table S1: Acquired targets of VC (vitamin C), CC (curcumin) and GA (glycyrrhizic acid)., Table S2: GO (Gene ontology) enrichment results from DAVID Bioinformatics Resources 6.8 (https://david.ncifcrf.gov/tools.jsp), including biological process (BP), cell component (CC) and molecular function (MF). Table S3: KEGG pathways enrichment results from ClueGo (integrates the latest KEGG database).
